# Brain hypoxia caused by respiratory obstruction which should not be forgotten in COVID-19 disease

**DOI:** 10.55730/1300-0144.5489

**Published:** 2022-06-18

**Authors:** Aleksandr URAKOV, Nikita MUHUTDINOV, Ilnur YAGUDIN, Darya SUNTSOVA, Milena SVETOVA

**Affiliations:** 1 Department of General and Clinical Pharmacology, Izhevsk State Medical Academy, Izhevsk, Russia; 2 Department of Modeling and Synthesis of Technological Processes, Udmurt Federal Research Center Ural Branch of the Russian Academy of Sciences, Izhevsk, Russia

**Keywords:** COVID-19, respiratory obstruction, pus, sputum, mucus, sodium peroxide solution

**To the Editor,**

We have read the article “COVID-19 and Sepsis” written by Zeliha Koçak Tufan et al. in the Turkish Journal of Medical Sciences with great interest (1). We appreciated this work and noted what fruitful work the authors did when writing this article. In fact, the number of cases and deaths caused by COVID-19 is increasing and at this point, the entire pathogenesis has not yet been studied and there is no effective therapy that could tip the scales in favor of recovery and avoid complications and death. We fully agree that one of the serious and significant complications that COVID-19 causes is cytokine storm and sepsis [1]. However, we want to note that the article pays little attention to the detailed breakdown of the formation and composition of sputum, pus, and mucus produced in the air space of the airways. Since we directly believe that they cause respiratory obstruction, and prevent the passage of air through the bronchi and alveoli, thereby disrupting gas exchange, which leads to cerebral hypoxia. Nowadays, artificial lung ventilation (ALV) machines with increased oxygen content in the inhaled air are used almost all over the world to prolong the life of critically ill patients with COVID-19 [2]. But over time, the bronchial and alveolar spaces are filled with sputum, pus, and mucus to the point of complete airway obstruction, which leads to hypoxia and death. Consequently, this method is not capable of prolonging the patient’s life for a long time. In fact, one solution that currently exists worldwide and contributes to the elimination of hypoxia is the use of extracorporeal membrane oxygenation (ECMO). However, this method is very expensive and limited in application, since the number of patients is very high, and funding of hospitals is limited. Therefore, it is impossible to provide this method to all seriously ill patients [3]. As practice shows, ECMO is only one way to prolong the life of patients and not the solution of the problem. Based on this, we highlight one of the problems that needs to be solved as soon as possible-it is the restoration of airway patency and elimination of hypoxia of the patient’s brain [4]. At the moment, there are a number of methods in which proposed oxygenation using a solution of hydrogen peroxide, which can be administered orally in the form of a drink carbonated with oxygen, and in the form of injections in the “right place” [4–6]. Therefore, it is necessary to change the approach and look for new drugs, as well as other ways to influence the links in the pathogenesis of the disease caused by COVID-19.
